# Etiological analysis of virus, mycoplasma pneumoniae and chlamydia pneumoniae in hospitalized children with acute respiratory infections in Huzhou

**DOI:** 10.1186/s12985-020-01380-4

**Published:** 2020-08-01

**Authors:** Min Gao, Xing Yao, Wei Mao, Cuifen Shen, Zongxin Zhang, Qiuling Huang, Dawei Cui, Haiyan Sun, Weihua Zou

**Affiliations:** 1 Department of Clinical Laboratory, Huzhou Central Hospital, Affiliated Central Hospital Huzhou University, Huzhou, 313000 China; 2 Hepatobiliary and Pancreatic Surgery, Huzhou Central Hospital, Affiliated Central Hospital Huzhou University, Huzhou, 313000 China; 3 Department of Respiratory Diseases, Huzhou Central Hospital, Affiliated Central Hospital Huzhou University, Huzhou, 313000 China; 4 Department of Pediatrics, Huzhou Central Hospital, Affiliated Central Hospital Huzhou University, Huzhou, 313000 China; 5 Department of Blood Transfusion, the First Affiliated Hospital, College of Medicine, Zhejiang University, Hangzhou, 310003 China; 6 Department of Clinical Laboratory, Shaoxing Second Hospital, Shaoxing, 312000 Zhejianeg Province China

**Keywords:** Acute respiratory infections, Hospitalized children, Etiology, Huzhou

## Abstract

**Background:**

Acute respiratory infections are a common disease in children with high mortality and morbidity. Multiple pathogens can cause acute respiratory infections. A 2-year survey of hospitalized children was conducted to understand the epidemic situation, seasonal spread of pathogens and the improvement of clinical diagnosis, treatment and prevention of disease in Huzhou, China.

**Methods:**

From September 2017 to August 2019, 3121 nasopharyngeal swabs from hospitalized children with acute respiratory infections were collected, and real-time PCR was used to detect various pathogens. Then, pathogen profiles, frequency and seasonality were analyzed.

**Results:**

Of the 3121 specimens, 14.45% (451/3121) were positive for at least one pathogen. Of the single-pathogen infections, RSV (45.61%, 182/399) was the most frequent pathogen, followed by PIVs (14.79%, 59/399), ADV (14.54%, 58/399), MP (10.78%, 43/399), and IAV (5.26%, 21/399). Of the 52 coinfections, RSV + PIVs viruses were predominantly identified and accounted for 40.38% (21/52) of cases. RSV was the most frequent pathogen in all four groups. The highest positive rate of the pathogens occurred in the winter (21.26%), followed by autumn (14.98%), the summer (14.11%) and the spring (12.25%).

**Conclusion:**

Viruses are the main pathogens in hospitalized children with acute respiratory infections in Huzhou city, Zhejiang Province, China. Among the pathogens, RSV had the highest detection rate, and MP is also a common pathogen among children with acute respiratory infections. This study provided a better understanding of the distribution of pathogens in children of different ages and seasons, which is conducive to the development of more reasonable treatment strategies and prevention and control measures.

## Background

Acute respiratory infections (ARIs) which can cause many deaths every year globally, particularly in pediatrics, are a common public safety threat and lead to high mortality and morbidity worldwide, especially in developing countries [1–3]. Many pathogens can cause ARIs, in addition to bacteria, many pathogenic microorganisms are difficult to isolate and culture. Including some viruses that are generally considered to be the main cause of the disease [4, 5], such as influenza A virus (IAV), respiratory syncytial virus (RSV), influenza B virus (IBV), adenovirus (ADV), parainfluenza 1, 2 and 3 (PIVs), and human rhinovirus (HRV), several new viruses, such as human metapneumovirus (hMPV) [6–8], human coronaviruses (HCoV)-NL63, 229E, OC43, and HKU1, human bocavirus (HBoV), mycoplasma pneumoniae (MP) and chlamydia pneumoniae (CP), have been discovered in human respiratory tract specimens with ARIs.

Currently, there are only a few available vaccines to prevent respiratory virus infections. Therefore, to understand the patient’s condition, treatment strategy, and prevention and control strategies, we must identify the patient’s pathogen. The rapid development of molecular diagnostic technologies, such as real-time polymerase chain technology [9] that can simultaneously perform nucleic acid amplification in numerous microorganisms. These advances allow for the reassessment of the effects of various breath pathogens.

China has an area of 9.6 million square kilometers, and the climate varies greatly from place to place. So far, there have been many epidemiological reports about ARIs such as Shenzhen and Gansu [1, 10]. The pathogen composition of ARIs is geographically diverse and related to the local epidemic status and climatic conditions [11, 12]. However, there is no applicable research about hospitalized children in Huzhou city, which is located in northern Zhejiang Province, China, the northern subtropical monsoon climate zone. To understand the etiological profile of viruses, MP and CP of inpatient children with ARIs in urban and surrounding areas, We participated in a national research program on respiratory infections to understand the etiological characteristics of respiratory infections in hospitalized children in Huzhou.

## Methods

### Patients and clinical samples

The study was conducted from September 2017 to August 2019 in Huzhou Central Hospital, Huzhou, Zhejiang Province. A total of 3121 nasal and throat swabs (NTS) were collected from hospitalized children diagnosed with ARIs. Patients were admitted according to the following criteria: (1) at least one of the following manifestations of acute infection: fever (temperature ≥ 37.5 °C), chills and abnormal white blood cell (WBC) differentials, leukocytosis (WBC count more than 10,000/mL) or leukopenia (WBC count less than 4000/mL); and (2) at least one of the following signs/symptoms of respiratory tract infection: cough, shortness of breath, sputum production, tachypnea, lung examination abnormalities (crackles or wheeze), and chest pain [13]. NTS were collected by nurses who have undergone rigorous training and assessment and kept in the transport medium and stored at − 80 °C prior to analysis. This study was approved by the Ethics Committee of Huzhou Center Hospital.

### Molecular detection of respiratory pathogens

Nucleic acid extraction was performed in strict accordance with the instructions of the Geneaid kit. According to the manufacturer’s instructions, we used real-time polymerase chain reaction (PCR), or real-time RT-PCR assay from Huirui Bio-Tech Co., Ltd., Shanghai, China to detect the nucleic acids.

All specimens were tested for IAV, RSV, IBV, ADV, PIV1–3, HRV, hMPV, HCoV, HBoV, MP and CP.

### Statistical analysis

Statistical analyses were conducted using SPSS 23.0 (SPSS Inc. Chicago, IL, USA). For comparison of categorical data, a chi-square test or Fisher’s exact test was used. *P*-value < 0.05 was considered to be statistically significant.

## Results

### Patient grouping and division by season

A total of 3121 children aged 7 days to 14 years after birth were divided into 4 groups: (1) 414 cases in the infants group (newborn ~ 1-year-old); (2) 969 cases in the toddlers group (> 1 ~ 3 years old); (3) 987 cases in the preschoolers group (> 3 ~ 6 years old); and (4) 751 cases in the schoolchildren group (7 ~ 14 years old). According to the seasons, all the children were divided into four groups: (1) 759 cases in the spring (March to May), 751 cases in the summer (June to August), 614 cases in the autumn (September to November), and 997 cases in the winter (December to February).

### Pathogen etiologies of ARIs in children

**Fig. 1:**
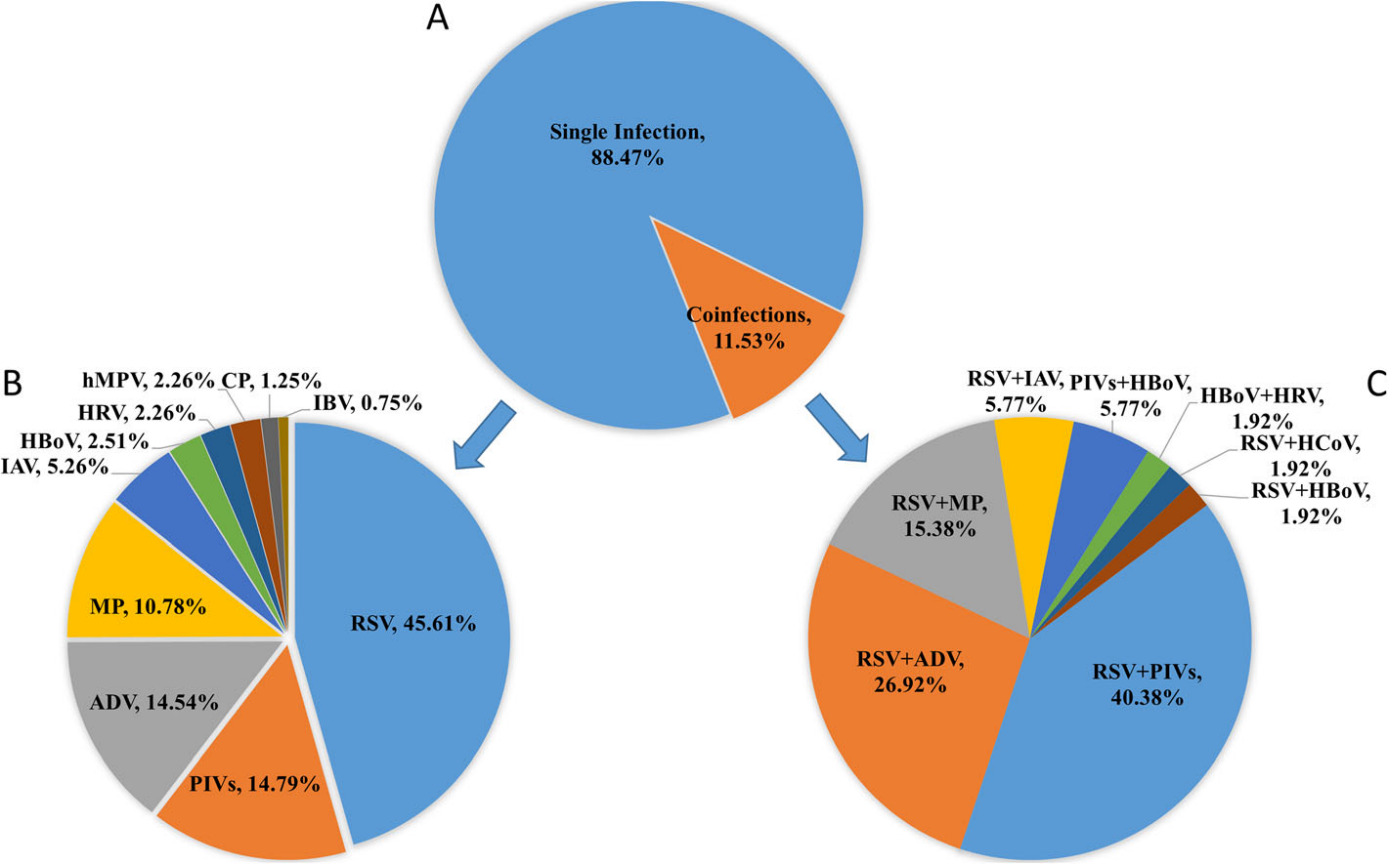
Identification of virus, MP and CP based on 451 pathogen-positive hospitalized children with ARIs. **a** Single infection and coinfections. In our study, the single-pathogen infection rate of ARIs in hospitalized children reached 88.47%. **b** Distribution of the 399 cases with single-pathogen infection. RSV was the most frequent pathogen, identified in 45.61% of the cases, followed by PIVs (14.79%), ADV (14.54%), MP (10.78%), IAV (5.26%), and other pathogens that were identified in under 5.0% of the samples. **c** Distribution of different combinations of the 52 cases with pathogen coinfections. RSV + PIVs viruses were predominantly identified and accounted for 40.38% cases, followed by RSV + ADV (26.92%), RSV + MP (15.38%), RSV + IAV (5.77%), PIVs+HBoV(5.77%), HBoV+HRV (1.92%), RSV + HCoV (1.92%) and RSV + HBoV (1.92%)

A total of 3121 clinical samples were collected from September 2017 to August 2019 in hospitalized children with ARIs. Of them, 451 specimens 14.45% (451/3121) were positive for at least one pathogen, a total of 403 cases were positive for the virus, with a positive rate of 12.91%, additionally, 56 cases were detected by MP and CP, with a positive rate of 1.80%. Single infections accounted for 88.47% (399/451) of cases in all positive patients. Coinfections were observed in 11.53% (52/451) of cases. Of the single pathogen infections, RSV was the most frequent pathogen, identified in 45.61% (182/399) of the cases, followed by PIVs (14.79%, 59/399), ADV (14.54%, 58/399), MP (10.78%, 43/399), IAV (5.26%, 21/399), and other pathogens that were identified in under 5.0% of the samples. Of the 52 coinfections, RSV + PIVs viruses were predominantly identified and accounted for 40.38% (21/52) of cases, followed by RSV + ADV (26.92%, 14/52), RSV + MP (15.38%, 8/52), RSV + IAV (5.77%, 3/52), PIVs+HBoV (5.77%, 3/52), HBoV+HRV (1.92%, 1/52), RSV + HCoV (1.92%, 1/52) and RSV+ HBoV (1.92%, 1/52) (Fig. 1). No other types of coinfections were found in this study.

### Demographic data of the hospitalized children admitted with ARIs

The distribution of the pathogens in the four age groups and different genders is shown in Table 1. The overall detection rate decreased with the increase of the age of enrolled children infected by single pathogen. The highest proportion was observed in the infants group (18.84%), and the proportion were 13.00% in the toddlers group, 11.45% in the preschoolers group and 10.92% in the schoolchildren group. There were significant differences in the detection rates of pathogens among the four groups (χ^2^ = 17.583, *p* = 0.001). The detection rates of the coinfections in the four groups were 2.42%, 1.34%, 2.13%, and 1.07% with increasing age, respectively, and there was no significant difference among the four groups. The detection rates by gender were 13.69% in males and 11.62% in females. There was no difference between males and females (χ^2^ = 2.947, *p* = 0.086). The coinfection rates were 1.65% and 1.68% in males and females, respectively, and there was no difference in different genders (χ^2^ = 0.003, *p* = 0.953).

**Table 1:** Demographic data of the hospitalized children admitted with ARIs

Variable	Total *N* = 3121 (%)	Single infection *N* = 399 (%)	coinfections *N* = 52 (%)
Age groups (years)			
Infants	414 (13.26%)	78 (18.84%)	10 (2.42%)
Toddlers	969 (31.05%)	126 (13.00%)	13 (1.34%)
Preschoolers	987 (31.62%)	113 (11.45%)	21 (2.13%)
Schoolchildren	751 (24.06%)	82 (10.92%)	8 (1.07%)
Total	3121	399 (12.78%)	52 (1.67%)
χ^2^ (P)		17.583 (0.001)	4.980 (0.173)
Gender			
Male	1753	240 (13.69%)	29 (1.65%)
Female	1368	159 (11.62%)	23 (1.68%)
χ^2^ (P)		2.947 (0.086)	0.003 (0.953)

The overall detection rate decreased with the increase of the age of enrolled children infected by single pathogen. There were significant differences in the detection rates of pathogens among the four groups (χ^2^ = 17.583, *p* = 0.001), while there was no significant difference in the coinfections among the four groups. There was no difference in different genders in single infection and coinfections

### Distribution characteristics of pathogens in different age groups

**Fig. 2:**
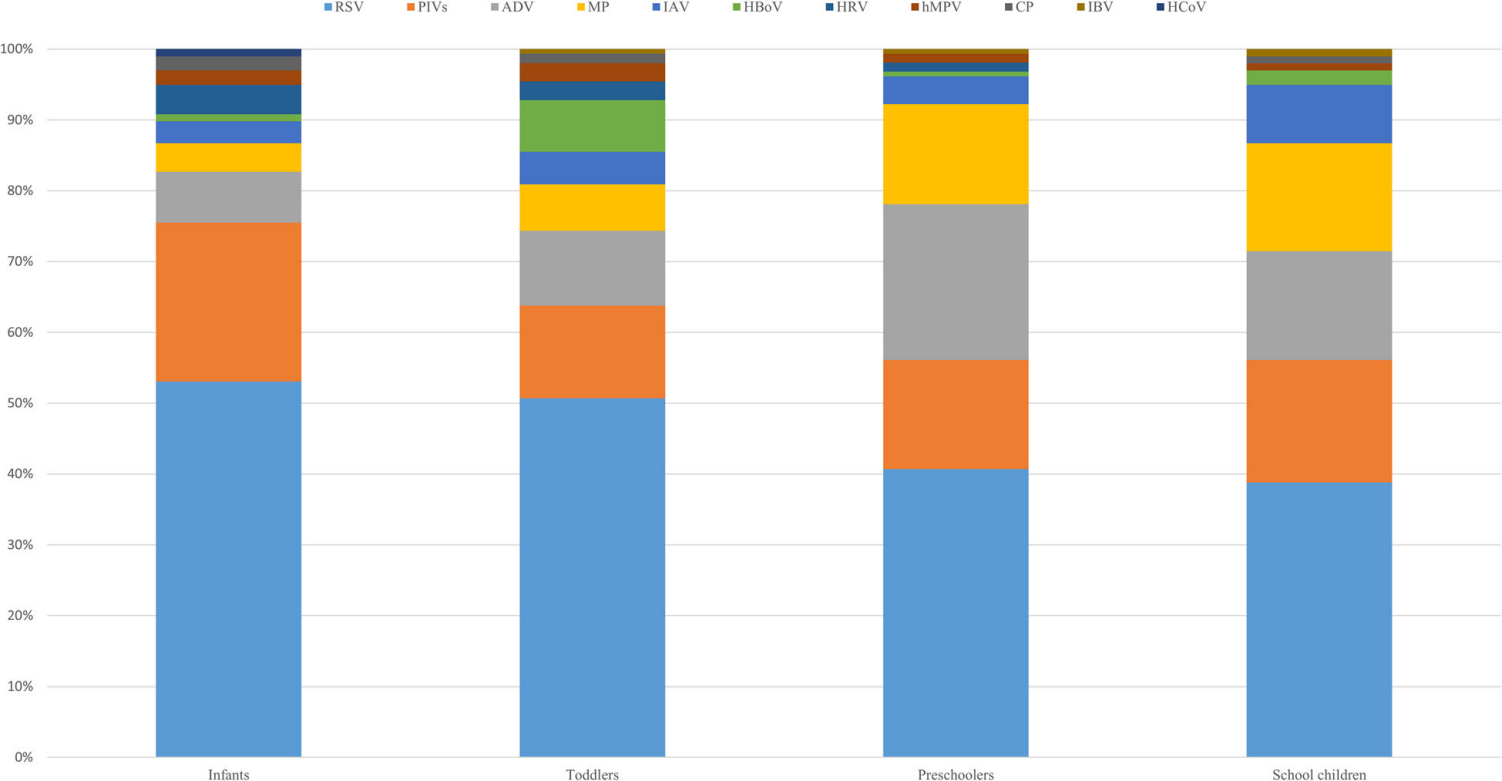
Distribution characteristics of pathogens in different age groups, In the figure, the abscissa indicated the four age groups and the ordinate indicated the detection rate of pathogens. In all four groups, we found that although the predominant pathogens are different, RSV had the highest detection rate in each group

In all four groups we found that although the predominant pathogens are different, but RSV had the highest detection rate in each group. However, as age increased, the detection rate decreased, while MP infection rates increased. In the infants group, PIVs (22.45%) were the second-most prevalent pathogens, followed by ADV (7.14%), MP (4.08%), HRV (4.08%), IAV (3.06%), hMPV (2.04%), CP (2.04%), HBoV (1.02%) and HCoV (1.02%). In the toddlers group, PIVs (13.16%) were the second-most prevalent pathogens, followed by ADV (10.53%), HBoV (7.24%), MP (6.58%), IAV (4.61%), HRV (2.63%), hMPV (2.63%), CP (1.32%) and IBV (0.66%). In the preschoolers group, ADV (21.94%) was the second-most prevalent virus, followed by PIVs (15.48%), MP (14.19%), IAV (3.87%), HRV (1.29%), hMPV (1.29%), and IBV (0.65%). In the schoolchildren group, PIVs (17.35%) were the second-most prevalent virus, followed by ADV (15.31%), MP (15.31%), IAV (8.16%), HBoV (2.04%), hMPV (1.02%), CP (1.02%) and IBV (1.02%) (Fig. 2).

### Seasonal distribution of different pathogens

**Fig. 3:**
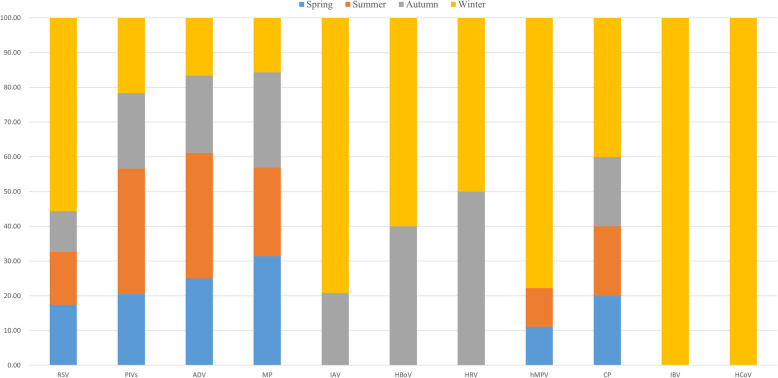
Seasonal distribution of different pathogens. In the figure, the abscissa represented different pathogens and the ordinate represented the detection rate of pathogens in different seasons. Respiratory pathogens can be detected throughout the year, but the rate was not distributed equally during different seasons. Most pathogens have the highest detection rate in winter, such as RSV, IAV, HRV, etc., while PIVs and ADV have the highest detection rate in summer

Respiratory pathogens can be detected throughout the year, but the rate was not distributed equally during different seasons in Huzhou city, Zhejiang Province (Fig. 3). The highest positive rate occurs in the winter (21.26%), followed by the autumn (14.98%), summer (14.11%) and spring (12.25%). Among the four major pathogens (RSV, MP, ADV and PIVs), RSV was most detected in the winter (55.65%), ADV and PIVs were most detected in the summer (36.11 and 36.14%), while MP can be detected in all four seasons, but the highest detected occurred in the spring (31.37%), followed by the autumn (27.45%), summer (25.49%), and winter (15.69%). The remaining pathogens (IAV, HBOV, HRV, and CP) were detected only in the autumn and winter, hMPV was mostly detected in the winter (77.78%), and IBV and HCoV were only detected in the winter.

### The positive rates of viruses, mycoplasma and chlamydia in the four age groups

With increasing age, the overall MP positive rate showed an upward trend, while CP had the opposite trend, but there was no significant difference among the four groups (χ^2^ = 6.124, *p* = 0.106; χ^2^ = 4.234, *p* = 0.132). Further analysis found that if the sample were divided into groups of children < 6 years old and > 6 years old, there was a significant difference (χ^2^ = 6.144, *p* = 0.013) in the MP positive rate between the two groups, while the CP difference was still no significant (χ^2^ = 1.339, *p* = 0.247). As age increased, the detection rate of viruses decreased, and there was a statistically significant difference among different age groups (χ^2^ = 28.735, *p* = 0.000) (Table 2).

**Table 2:** Mycoplasma-, chlamydia- and virus-positive rates in different age groups

Pathogen	Infants (414)	Toddlers (969)	Preschoolers (987)	Schoolchildren (751)	χ2	P
MP	4 (0.97%)	10 (1.03%)	22 (2.03%)	15 (2.00%)	6.124	0.106
CP	2 (0.48%)	2 (0.21%)	0 (0.00%)	1 (0.13%)	4.234	0.132
virus	92 (22.22%)	140 (14.45%)	133 (13.48%)	82 (10.92%)	28.735	0.000

With increasing age, the overall MP positive rate showed an upward trend, while CP had the opposite trend, but there was no significant difference among the four groups. As age increased, the detection rate of viruses decreased, and there was a statistically significant difference among different age groups

## Discussion

To our knowledge, this is the first comprehensive virus, MP, and CP etiology study of hospitalized pediatric patients with ARIs, including newly identified respiratory viruses, in Huzhou. ARIs account for approximately 20% of premature deaths in infants by systematic analysis [14]. Prior studies have noted the etiology and epidemiology of hospitalized ARIs patients, including children and/or adults worldwide [15–17], but the study of inpatient in children with ARIs is more limited.

We conducted a large study from 2017 to 2019 to assess the regional common pathogens infection pattern in children in Huzhou, China. General respiratory pathogens, including RSV, ADV, PIVs, IAV, IBV, MP, and CP, as well as newly identified viruses, such as HBoV, HCoV, and hMPV, were detected by PCR, then, the age and gender distribution, infection frequency, and seasonality of the respiratory infectious pathogens were analyzed.

In 3121 children, the detection rate of pathogens was 14.45% (451/3121), and a total of 403 cases were positive for the virus, the positive rate was 13.07%. Wang H, et al. had reported the nearly result in Shenzhen [16], but it was lower than in 22 provinces of China (36.6%) and Shandong (35.75%) [7, 13]. The overall infection rate decreased with the increase of children’s age, these results suggested that pathogens in ARIs were closely associated with patient’s age which affects exposure opportunities to viruses and immune status, the result is consistent with the previous report [18]. The single infection rate in infants is as high as 18.84%. The reason for this phenomenon may be that immunity gradually increases with the child’s growth. Infants with weak immunity, rapid disease changes, and high detection rates of pathogens should be given high clinical attention.

This study found that some of the children’s respiratory NTS detected two pathogenic nucleic acids, RSV + PIVs and RSV + ADV infection, which were ranked in the top two. There was no significant difference among the four groups. The detection rate was 2.42% in the infants’ group, while the schoolchildren group was 1.07%. As age increased, coinfection tended to decrease. Chen J, et al. [19] have reported a similar result in Chengdu. Until now, there has been no consistent opinion on the cause of coinfection [20, 21] and the impact on the severity of the disease is still controversial [22]. The results of Chen YW, et al. [23] in northern Taiwan and Asner SA, et al. [24] showed that there was no difference in clinical disease severity between viral coinfections and single respiratory infections. The reason for coinfection may be that children’s immune systems are not mature enough and susceptible to infection by a variety of pathogens, so young children should pay more attention. Then we will study whether coinfection is related to the severity of the disease, complications, and prognosis.

Our study showed that RSV was the most frequently detected in hospitalized children of ARIs, but as age increased, the positive rate decreased, which was consistent with previous studies in Shenzhen, Shandong, Chengdu, Taiwan, Turkey and Russia [2, 7, 19, 23, 25, 26], followed by PIVs, ADV, MP, IAV, and other pathogens that were identified in under 5.0% of the sample. Several cities, such as Suzhou and Shanghai within 150 km of Huzhou, all also have been found that the virus detection rate tends to decline with the increase of age, while the detection rate of MP is the opposite. Shanghai [27] results showed that the detection rate of RSV (33.59%), PIVs (13.28%) and ADV (3.97%) ranked the top three in viral infections, The study in Suzhou [28] from 2005 to 2011 also found that RSV (35.51%), hMPV (10.71%) and PIVs (5.84%) ranked the top three in viral infections. We found that the MP detection rate in both places was higher than that in Huzhou, It may be mainly due to the following reasons: Firstly, the MP infection rate was greatly affected by seasonal weather and varied greatly from year to year. Secondly, they used a combination of PCR and ELISA to detect MP in the Suzhou study, which also increased the detection rate. Thirdly, the patients studied in Shanghai were mainly with lower respiratory tract infections. We also founded that the detection rate of hMPV was significantly higher in Suzhou than ours, mainly because the number of infants (51.8%) included in the Suzhou study was relatively large, while hMPV mainly infected infants. The detection rate of RSV was 45.61% in our research which was higher than 16.02% in Shandong province [7], 33.70% in Chengdu province [19] in other regions of China. RSV may cause annual epidemics worldwide because of variability in the virus [29]. The prevalence of RSV had been reported to be related to alternating cycles of multiple genotypes and changes in G protein [30]. Both temporal and geographic clustering of particular may occur [31]. In summary, the detection of respiratory infection pathogens is greatly influenced by climate and varies from place to place. Our research showed that the detection rate of RSV was high, probably because the time we studied was in the RSV popular years and climatic conditions were conducive to the spread of RSV.

In our study, respiratory pathogen infections had major seasonal changes, most of which occurred in the winter and were lowest in the spring. Similar results were reported in other studies [18, 32], which is different from Shenzhen and Shandong [2, 7]. The difference in seasonal pathogen detection is affected by a variety of factors, among which climate is an important factor, such as the time of sunlight exposure, temperature, humidity and so on, which change the duration of the virus in the environment [33]. The RSV detection rate was higher in the winter and spring, which was consistent with previous studies [34, 35] and different from Shenzhen [2], while MP can be detected in all seasons, it is slightly higher in the spring and autumn but lowest in the winter. PIVs promote a variety of clinical manifestations and result in asymptomatic pneumonia [36]. With the exception of the preschooler group, the positive rate of PIVs was second in each group. It had the highest detection rate in summer and can be detected all year round, unlike Chengdu [19]. Such as IAV, HCoV and so on have the highest detection rate in the winter. We found the characteristics of the peak time of pathogen infection showed certain homogeneity between Huzhou, Shanghai and Suzhou [28], RSV peaked in winter, in contrast, higher levels of MP and PIVs detected relatively warm weather, lower in winter in Shanghai [27]. Wei J, et al. [28] also found RSV exhibited notable seasonal distributions, peaking in winter months, ADV and PIVs were more frequently detected in summer in Suzhou. These results indicate the geographical diversity and climate of the Taihu Lake region could contribute to seasonal variations of pathogens that cause ARIs.

MP can cause upper and lower respiratory tract infections, is one of the important pathogens causing atypical pneumonia, and easily causes outbreaks in children. Our research indicated that the detected rate of MP was higher in children over 6 years old than the lower age groups, while the virus is the opposite. Therefore, different treatment and prevention measures should be formulated for children of different age groups.

Our research still left much to be desired. First, the sample volume was not sufficient, with only 3121 patients and 451 positive cases. Second, the case collection did not last long enough. More large-scale cases that last longer can lead to a better assessment of the seasonal distribution of pathogens throughout the year and the detection rate in the four groups.

## Conclusions

We first revealed many pathogens, especially RSV, PIVs, ADV, and MP, play an important role in hospitalized children with ARIs in Huzhou city, Zhejiang Province, China. At the same time, we also revealed the distribution characteristics of pathogens in different ages and seasons, which is conducive to the development of more effective treatment strategies and prevention and control measures in hospitalized children.

## Data Availability

Not applicable.

## Abbreviations

ARIs: Acute respiratory infections
IAV: Influenza A virus
RSV: Respiratory syncytial virus
IBV: Influenza B virus
ADV: Adenovirus
PIVs: Parainfluenza 1, 2 and 3
HRV: Human rhinovirus
hMPV: human metapneumovirus
HCoV: Human coronaviruses
HBoV: Human bocavirus
MP: Mycoplasma pneumoniae
CP: Chlamydia pneumoniae
NTS: Nasal and throat swabs
WBC: White blood cell
PCR: Polymerase chain reaction
